# Endemics Versus Newcomers: The Ladybird Beetle (Coleoptera: Coccinellidae) Fauna of Gran Canaria

**DOI:** 10.3390/insects11090641

**Published:** 2020-09-18

**Authors:** Jerzy Romanowski, Piotr Ceryngier, Jaroslav Vĕtrovec, Marta Piotrowska, Karol Szawaryn

**Affiliations:** 1 Institute of Biological Sciences, Cardinal Stefan Wyszyński University, Wóycickiego 1/3, 01-938 Warsaw, Poland; p.ceryngier@uksw.edu.pl; 2 Buzulucká, 1105 Hradec Králové, Czech Republic; JerryVetrak@seznam.cz; 3 Faculty of Biology and Environmental Sciences UKSW, ul. Wóycickiego 1/3, PL-01-938 Warsaw, Poland; mpmartapiotrowska@gmail.com; 4 Museum and Institute of Zoology, Polish Academy of Sciences, Wilcza 64, 00-679 Warsaw, Poland; k.szawaryn@gmail.com

**Keywords:** biodiversity, Canary Islands, alien species, new records

## Abstract

**Simple Summary:**

Many plants and animals that live in the Canary Islands belong to the so-called endemic species, i.e., they do not occur outside of this particular region. Several other species have a slightly wider geographical distribution, apart from the Canaries, which also includes some islands of the nearby archipelagos, such as Madeira or the Azores, or the northwestern periphery of Africa. Here, we call such species subendemics. However, the Canary Islands have recently been colonized by a substantial number of immigrants from more or less remote areas. In this paper, based on our field survey and previously published data, we analyzed the fauna of the ladybird beetles (Coccinellidae) of Gran Canaria, one of the central islands of the archipelago. Among 42 ladybird beetle species so far recorded on this island, 17 (40%) are endemics and subendemics, and 21 (50%) probably arrived in Gran Canaria relatively recently, i.e., in the 20th and 21st century. Of those newcomers, there are Australian, American, African, and European species. The nonnative ladybird species may pose a threat to the unique ladybird communities from Gran Canaria and other islands of the archipelago.

**Abstract:**

Research on the fauna of beetles (Coleoptera) of the Canary Islands has a long tradition, which enables tracking changes in their species composition and arrival of new species. In this paper, we provide new faunistic data on the ladybird beetles (Coccinellidae) recorded on Gran Canaria, one of the central islands of the archipelago, and then analyze available information on the Gran Canarian ladybird fauna from geographical and historical points of view. The field survey resulted in recording 1402 ladybird individuals belonging to 30 species. Ten of these species were new to Gran Canaria and three of them, *Chilocorus bipustulatus* (Linnaeus), *Nephus bisignatus* (Boheman), and *Nephus ulbrichi* Fürsch, had not previously been reported to be on any of the islands of the Canarian archipelago. *Tetrabrachys tinerfensis* (Hodgson) is synonymized with *T. deserticola* (Wollaston). Our survey and literature reports allowed us to recognize 42 species of Coccinellidae so far recorded on Gran Canaria. Seventeen of them (40%) belonged to the Canarian endemic and subendemic species, and 21 (50%) were newcomers and presumed newcomers. Colonization of Gran Canaria and other islands of the archipelago by ladybird species of various origins seems to be a frequent phenomenon that may pose a threat to the unique communities of the native Canarian species.

## 1. Introduction

A substantial portion of the Canarian biodiversity is composed of endemic species. For example, the endemism rate among the terrestrial arthropods is estimated to be around 38% [1,2]. The subject of our study, the Coleopteran family Coccinellidae, has about 50 recorded species in the Canaries [3,4,5], and 14 of them (28%) are considered to be endemic species [3]. However, many of the Canarian ladybirds have only recently arrived on the archipelago. Several such species were listed by Oromí et al. [3] and the next two were added by Suárez et al. [5] and Romanowski et al. [4].

The first published data on the Coccinellidae of the Canary Islands came from the early 19th century [6,7,8]. Then, significant contributions to the knowledge of the Canarian ladybirds were made by Wollaston [9,10], Uyttenboogaart [11,12], Korschefsky [13], Lindberg [14], and Fürsch [15]. More recently, further reports were provided by various authors, and the available data were summarized by Machado and Oromí [16], Eizaguirre [17], and Oromí et al. [3]. Despite this long history of studying ladybird fauna in the Canary Islands, the species composition on individual islands has not been sufficiently documented. Our recent surveys on Fuerteventura [4,18], Lanzarote [19], and El Hierro [20] allowed us to record many ladybird species not previously reported to be on those islands.

This paper concerns the Coccinellidae on another island of the archipelago, Gran Canaria. First, we provide new faunistic data, and then analyze available information on the Gran Canarian ladybird fauna from the geographical (distributional patterns) and historical (dates of records) points of view.

## 2. Study Area, Materials, and Methods

The Canary Islands lie in the northeast Atlantic Ocean near the African coast and are comprised of seven main islands and several islets. High biodiversity combined with diversification of the islands with respect to the size, age, landforms, and distance from the continent [1,2] make the archipelago an excellent system for biogeographical analyses.

**Figure 1:**
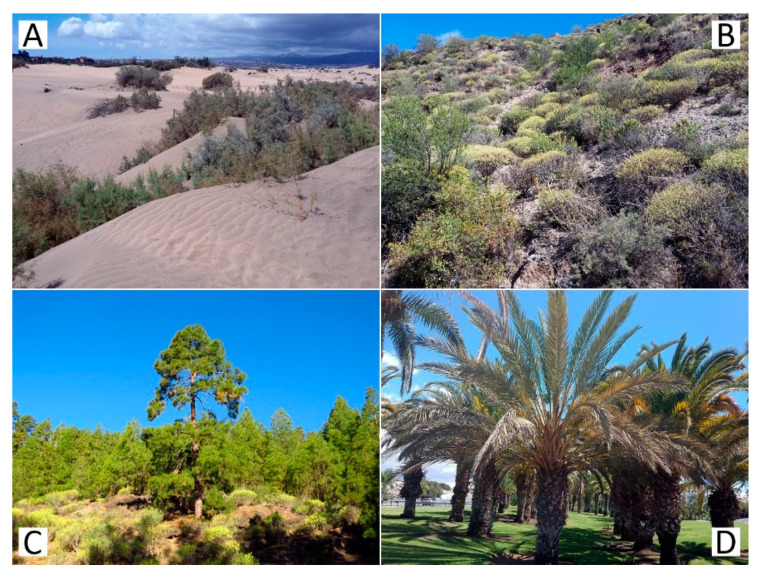
Some of the habitats surveyed in this study. (**A**) Dunes in Maspalomas; (**B**) Scrub vegetation with *Euphorbia* spp.; (**C**) Pine forest with *Pinus canariensis;* (**D**) Park vegetation in Maspalomas.

Gran Canaria is one of the central islands of the Canarian archipelago, located some 200 km from the African mainland. It was formed about 14.5 million years ago as a result of volcanic eruptions [2]. Of nearly circular shape, the island is elevated to 1950 m a.s.l. at its highest point (Pico de las Nieves) [21]. The vegetation of Gran Canaria includes a wide range of habitats, such as coastal dunes, scrub vegetation with *Euphorbia* spp., or pine forests with *Pinus canariensis* D. Smith. In anthropogenic habitats, decorative plants sustained by irrigation are cultivated (Figure 1).

### 2.1. Faunistic Survey

Ladybirds were recorded at 55 sites on Gran Canaria (Table 1) using standard collecting methods, such as beating tray, sweeping net, or direct observation. Although some of the caught ladybird individuals were released after their identification, each individual was noted. The majority of specimens were recorded by J. Romanowski and P. Ceryngier between 31 March and 6 April 2019. Some material collected earlier in 2001, 2015, 2016, and 2017 by F. Pavel, J. Krátký, and M. Piotrowska, was also used in this study. In the result section, we mention names of the collectors if they are different from J. Romanowski and P. Ceryngier. The voucher specimens are stored in the insect collection in the Institute of Biological Sciences, Cardinal Stefan Wyszyński University in Warsaw and in private collections of Jaroslav Větrovec (materials collected by F. Pavel and J. Krátký) and Marta Piotrowska. Unless specifically discussed, the nomenclature of Coccinellidae follows Kovář [22], and systematic arrangement follows Ślipiński [23] and Seago et al. [24].

### 2.2. Geographical and Historical Analysis of the Ladybird Fauna on Gran Canaria

On the basis of the literature data and results of this study, we compiled a list of all ladybird species recorded on Gran Canaria. Records of these species, both on Gran Canaria and other islands of the Canary archipelago, were allocated among the following three time periods: (1) 19th century (primarily the classical Wollaston’s [9,10] works); (2) first half of the 20th century (important contributions from Uyttenboogaart [11,12,25] and Lindberg [14], and several other reports); and (3) records after 1950.

Next, according to this historical arrangement of records and available data on the general distribution of the species recorded on Gran Canaria, we assigned each species to one of six groups within the following two rough categories: (1) endemic and subendemic species and (2) species with wider ranges. In the first category, we distinguished species endemic to Gran Canaria, species endemic to the Canary archipelago, and subendemic species. The latter category included species known both from the Canary Islands and some other islands of Macaronesia or from the Canary Islands and northwestern Africa. Among the species with wider ranges, we distinguished a group of non-endemic old inhabitants of the archipelago, presumed newcomers and alien species. According to our classification, the old inhabitants were widely distributed taxa already recorded in the Canary Islands in the 19th century. The presumed newcomers were not recorded in this period and, hence, probably arrived on the archipelago relatively recently. This group consisted of species that had spread naturally from the Mediterranean region, Africa, or other islands of Macaronesia, as well as those whose arrival in the Canary Islands associated with human activities such as trade. The last group (alien species) consisted of species coming from remote places in the world. They certainly or almost certainly arrived in the Canary Islands through the mediation of man. 

## 3. Results and Discussion

### 3.1. Faunistic Survey

Altogether, 1402 ladybird individuals (1327 adults, 72 larvae, and 3 pupae) belonging to 30 species were recorded in this study. Ten of the recorded species are new to Gran Canaria and three of them are new to the Canary Islands. Detailed data on all the recorded species are provided below. Morphological and anatomical details of several species of special interest (endemics and newcomers) are photographed.

**Microweiseinae Leng, 1920**

**Serangiini Pope, 1962**

***Delphastus catalinae* (Horn, 1895)**

**Material examined:** Las Palmas: 4.IV.2019, 1 ex. from *Phoenix canariensis* H. Wildpret; Maspalomas: 5.IV.2019, 1 ex. from *Nerium oleander* L.

**Distribution:** Native to North America but used outside of its native range as a biocontrol agent against whiteflies (Hemiptera: Aleyrodidae) [26]. Recorded on most of the islands of the Canarian archipelago, i.e., La Palma [27], La Gomera [17], Tenerife [17,28], Fuerteventura [18], and Lanzarote [19]. New to Gran Canaria.

**Coccinellinae Latreille, 1807**

**Chilocorini Mulsant, 1846**

***Chilocorus bipustulatus* (Linnaeus, 1758)**

**Material examined:** Cabo Verde: 3.IV.2019, 1 ex. (larva bred in the laboratory to adulthood).

**Distribution:** Widely distributed in the Palaearctic region including the Azores and Madeira [22], but not previously reported to be on the Canary Islands.

***Chilocorus canariensis*** **Crotch, 1874**

**Material examined:** Degollada de la Yegua: 29.I.2016, 1 ex. (leg. J. Krátký).

**Distribution:** Species endemic to the Canary Islands, reported to be on all islands of the archipelago [3].

**Figure 2:**
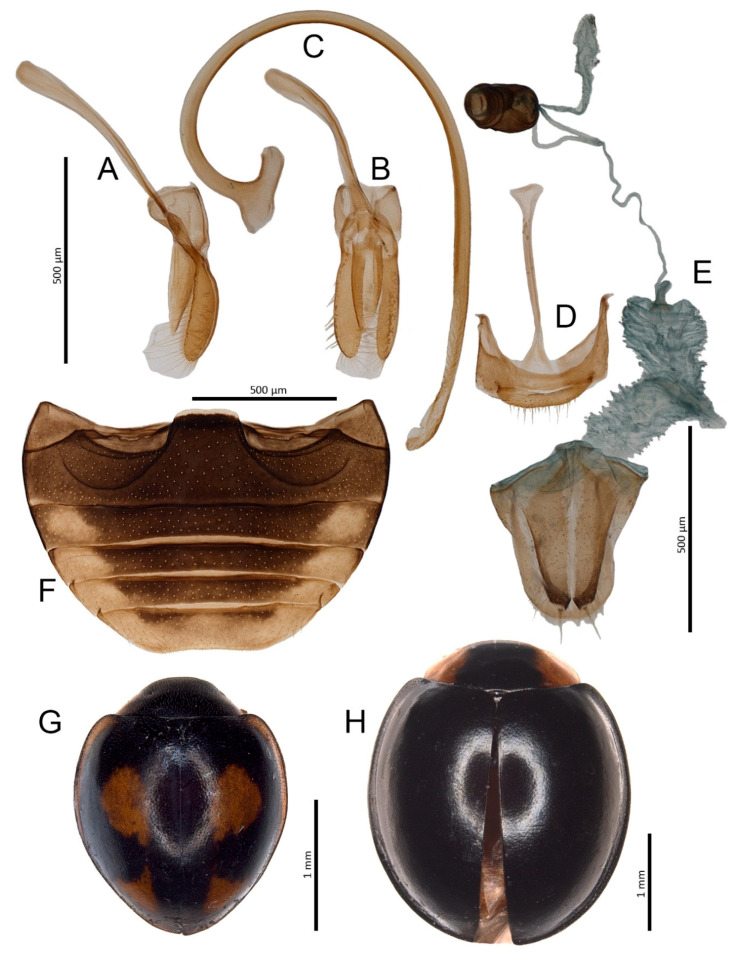
*Parexochomus bellus*. (**A**) Tegmen, lateral; (**B**) Tegmen, inner; (**C**) Penis, lateral; (**D**) Male genital segment; (**E**) Female genitalia; (**F**) Abdomen, male; (**G**) Habitus. *Parexochomus nigripennis*. (**H**) Habitus.

***Parexochomus bellus* (Wollaston, 1864)** (Figure 2A–G)

**Material examined:** Tufia: 18.II.2015, 1 ex. (leg. J. Krátký); Maspalomas: 21–23.VIII.2017, 3 exx. (leg. M. Piotrowska); 1.IV.2019, 8 exx. (6 imagines and 2 larvae) from *Launaea arborescens* (Batt.) Murb.; 5.IV.2019, 2 exx. from *N. oleander*.

**Distribution:** Endemic Gran Canarian species, until this study, only reported from the surroundings of Maspalomas [9,29] and Las Palmas [10,11]. Tufia, on the eastern coast of the island, is the third known locality of this species.

***Parexochomus nigripennis* (Erichson, 1843)**

**Material examined:** Tufia: 28.I.2016, 1 ex. (leg. J. Krátký); Las Palmas: 4.IV.2019, 1 ex. from *Hibiscus* sp.; Maspalomas: 1.IV.2019, 2 adults from *N. oleander*, 1 larva from *L. arborescens*; 5.IV.2019, 3 larvae from *N. oleander*.

**Distribution:** Widely distributed in the Mediterranean and Middle East countries, as well as in northwestern India, Pakistan and the Afrotropical region [22,30]. Known to be on all islands of the Canary archipelago except La Palma [3,17].

**Remarks:** All specimens collected in this study represent color form untypical for this species, i.e., with the pronotum predominantly black and with yellow lateral patches (Figure 2H). In typically colored *P. nigripennis* the pronotum is entirely yellow. Comparison of male and female genitalia of this form with those of typical form showed no differences.

**Coccidulini Mulsant, 1846**

***Cryptolaemus montrouzieri* Mulsant, 1853**

**Material examined:** Maspalomas: 21–22.VIII.2017, 2 exx. (leg. M. Piotrowska); Arucas, Las Palmas, Maspalomas, Pozo Izquierdo, Teror, Vecindario: 1–6.IV.2019, total of 54 exx. (47 adults, 7 larvae) collected from *N. oleander*, *Dracaena* sp., *Pistacia lentiscus* L., *Phoenix canariensis*, *Bougainvillea* sp.

**Distribution:** Australian species, widely used as a biological control agent and established throughout the warmer regions of the world [31], including all seven islands of the Canary archipelago [3,18,19].

**Figure 3:**
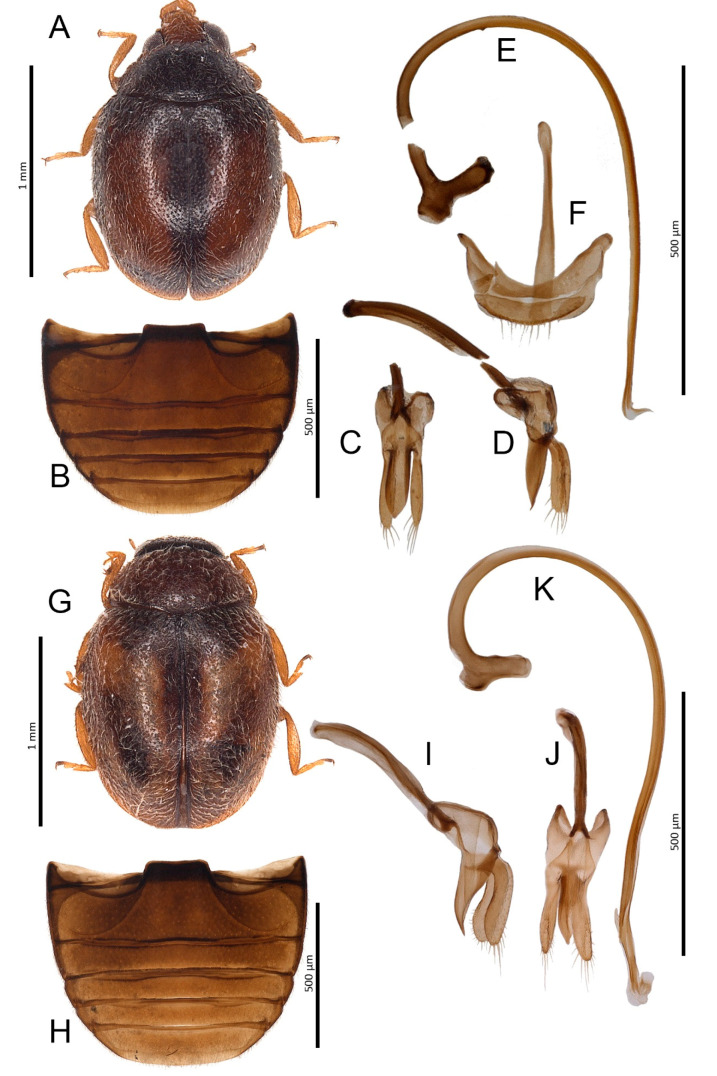
*Nephus bisignatus*. (**A**) Habitus; (**B**) Abdomen, male; (**C**) Tegmen, inner; (**D**) Tegmen, lateral; (**E**) Penis, lateral; (**F**) Male genital segment. *Nephus ulbrichi*. (**G**) Habitus; (**H**) Abdomen, male; (**I**) Tegmen, lateral; (**J**) Tegmen, inner; (**K**) Penis, lateral.

***Nephus (Bipunctatus) bisignatus* (Boheman, 1850)** (Figure 3A–F)

**Material examined:** Cruz de Tejeda: 16.XII.2015, 2 ♂♂.

**Distribution:** European species [22]. In Macaronesia previously reported to be on the Azores [15]. New to the Canary Islands.

**Remarks:** Both specimens of *N. bisignatus* that have been examined were chestnut brown in color which is probably teneral, while the typical coloration of mature beetles is black. Comparison of male genitalia of these specimens with those of typical form showed no differences.

***Nephus (Nephus) flavopictus* (Wollaston, 1854)**

**Material examined:** Ingenio, Barranco de Guayadeque: 18.II.2015, 1 ex. (leg. J. Krátký); Artenara: 29.I. 2016, 1 ex. (leg. J. Krátký); Lanzarote: 1.II.2016, 1 ex. (leg. J. Krátký); Arucas: 3.IV.2019, 2 exx.; Cementerio Santa Lucía: 31.III.2019, 1 ex.; Las Palmas: 4.IV.2019, 1 ex.; Maspalomas: 5.IV.2019, 1 ex.; San Felipe: 3.IV.2019, 2 exx.; Cabo Verde: 3.IV.2019, 2 exx.; Las Hojas: 2.IV.2019, 5 exx. Collected mostly from succulents, *Tamarix* sp., *Ficus sp.*, *N. oleander,* and *Hibiscus* sp.

**Distribution:** Macaronesian species, reported to be on the Azores [15,32], Madeira [15,33,34], and all the Canary Islands [3].

***Nephus* (*Nephus*) *incisus* (Har. Lindberg, 1950)**

**Material examined:** Las Palmas: 4.IV.2019, 5 exx. on *N. oleander*; Maspalomas: 5.IV.2019, 4 exx. from *Phoenix canariensis* and *N. oleander*; Pie de la Cuesta: 1.IV.2019, 1 ex. from *Bougainvillea* sp.; Vecindario: 6.IV.2019, 2 exx. from *Bougainvillea* sp. and *Hibiscus* sp.; Mirador El Mulato: 31.III.2019, 1 ex. from undetermined Fabaceae.

**Distribution:** Endemic Canarian species, known to be on all islands of the archipelago [3,18,19,20].

***Nephus* (*Nephus*) *ulbrichi* Fürsch, 1977** (Figure 3G–K)

**Material examined:** Cruz de Tejeda: 16.XII.2015, 5 exx.

**Distribution:** South European species [22], new to the Canary Islands.

***Rhyzobius litura* (Fabricius, 1787)**

**Material examined:** El Rincón Barranco de la Coruña: 20.II.2015, 1 ex. (leg. J. Krátký); Barranco de Santa Brígida: 21.II.2015, 1 ex. (leg. J. Krátký), 25.I.2016, 1 ex. (leg. J. Krátký); Barranco de los Cernícalos: 21.I.2016, 1 ex. (leg. J. Krátký); Fontanales: 3.IV.2019, 1 ex. on *Rubus* sp.

**Distribution:** Widely distributed in Europe and North Africa, and also reported to be from the Asiatic part of Turkey [22]. Reported to be on all islands of the Canary archipelago [3].

***Rhyzobius lophanthae* (Blaisdell, 1892)**

**Material examined:** Los Tiles de Moya: 25.I.2015, 1 ex. (leg. J. Krátký); Ingenio, Barranco de Guayadeque: 18.II.2015, 1 ex. (leg. J. Krátký); Barranco de los Cernícalos: 28.I.2016, 1 ex. (leg. J. Krátký); Acusa Verde: 30.I.2016, 1 ex. (leg. J. Krátký); Maspalomas: 21–22.VIII.2017, 2 exx. (leg. M. Piotrowska); Arucas, Cabo Verde, Cruz de San Antonio, Firgas, La Herradura, La Sorrueda, Las Palmas, Maspalomas, Mirador El Mulato, Pie de la Cuesta, Pozo Izquierdo, Presa de las Niñas, San Bartolomé, Santa Lucía de Tirajana: 31.III–5.IV.2019, total of 148 exx. (145 adults, 3 larvae) collected mostly from *Cycas* sp., *Phoenix canariensis*, *Dracaena* sp., *Euphorbia* sp., *Hibiscus* sp., and *Agave* sp.

**Distribution:** Species of Australian origin, introduced throughout the world for biocontrol purposes [35]. Known from all Canary Islands [3,17].

**Remark:** Although Kovář [22] placed this species in the genus Lindorus Casey, we followed Pope’s [36] synonymization of Lindorus with Rhyzobius Stephens, a decision that was also retained by Tomaszewska [35] in her revisionary work on Rhyzobius.

***Scymnus (Mimopullus) cercyonides* Wollaston, 1864**

**Material examined:** Mogán: 18.II.2015, 1 ex. (leg. J. Krátký); Santa Lucía de Tirajana: 2.II.2016, 1 ex. (leg. J. Krátký); Cruz de San Antonio: 1.IV.2019, 1 ex. from *Pinus canariensis*.

**Distribution:** Endemic Canarian species, reported to be on all islands of the archipelago except the easternmost Fuerteventura and Lanzarote [3].

***Scymnus (Pullus) canariensis* Wollaston, 1864**

**Material examined:** Ingenio, Barranco de Guayadeque: 18.II.2015, 2 exx. (leg. J. Krátký); Tufia: 18.II.2015, 4 exx. (leg. J. Krátký); Agaete: 23.II.2015, 8 exx. (leg. J. Krátký), 30.I.2016, 2 exx. (leg. J. Krátký); Barranco de los Cernícalos: 25.I.2016, 3 exx. (leg. J. Krátký); Artenara: 29.I.2016, 9 exx. (leg. J. Krátký); Cruz de Timagada: 1.II.2016, 1 ex. (leg. J. Krátký); Arucas, Ayacata, Barranco Hondo, Cabo verde, Cementerio Santa Lucía, Cruz de San Antonio, Cruz de Timagada, El Pocillo, Fontanales, Juncalillo, La Degollada, La Herradura, La Solana, La Sorrueda, Las Hojas, Las Palmas, Maspalomas, Mirador El Mulato, Mundo Aborigen, Pie de la Cuesta, Pozo Izquierdo, Presa de las Niñas, Rosiana, San Bartolomé, Santa Lucía de Tirajana, Tejeda, Vecindario, Vega de San Mateo: 31.III–6.IV.2019, total of 395 specimens (390 adult, 5 larvae) collected from various plants including, *Juniperus* sp., *N. oleander, Prunus dulcis* (Mill.) D.A.Webb, *Hibiscus* sp., *Phoenix canariensis*, *Pinus canariensis*, *Euphorbia* sp., *L. arborescens, Hedera* sp., *Agave* sp., *Olea europaea* L. and herbaceous vegetation.

**Distribution:***S. canariensis* has been recorded throughout the Canary Islands and considered to be endemic to the archipelago [3]. The recent reports from outside of this range (São Tomé and Príncipe and Senegal) [37] should be treated with caution, because they were based on an inventory of museum collections without examination of specimens.

***Scymnus (Pullus) medanensis* Eizaguirre, 2007**

**Material examined:** Maspalomas: 22.VIII.2017, 1 ♂ (leg. M. Piotrowska).

**Distribution:** Described from Tenerife [17]. Recently its presence on Fuerteventura [18] and Lanzarote [19] was documented. New to Gran Canaria.

***Scymnus (Pullus) subvillosus durantae* Wollaston, 1854**

**Material examined:** Las Palmas, Maspalomas, Pie de la Cuesta, Vecindario: 1–6.IV.2019, total of 66 specimens collected from *N. oleander*, *Hibiscus* sp., and *Bougainvillea* sp.

**Distribution:** Taxon of uncertain status, probably occurring in Macaronesia and western Africa, see [18]. Within the Canary Islands, previously recorded on La Palma, La Gomera, Tenerife, Fuerteventura, and Lanzarote [3,18,19]. New to Gran Canaria.

**Remarks:** Kovář [22] treated *S. durante* (sic!) as a synonym of *S. subvillosus*. However, Eizaguirre [17] emphasized the distinctiveness of the Canarian populations of *S. subvillosus*, giving them the rank of subspecies. We comply with this decision.

***Scymnus (Scymnus) nubilus* Mulsant, 1850**

**Material examined** Ayagaures: 17.XII.2015, 2 exx.; Arucas, La Herradura, Las Palmas, Maspalomas: 1–5.IV.2019, total of 37 exx. collected from *N. oleander*, *Ficus* sp., *Tamarix* sp., *Hibiscus* sp., *Pinus canariensis* and *L. arborescens*.

**Distribution** Widely distributed in the Mediterranean Basin and the Middle East, and also reported to be from the Afrotopical and Oriental regions [22]. Known from all Canary Islands except La Palma [3,18,19].

***Stethorus tenerifensis* Fürsch, 1987**

**Material examined:** Cueva Grande: 26.I.2016, 1 ex. (leg. J. Krátký); Arucas, Ayacata, Barranco Hondo, Cabo Verde, Cementerio Santa Lucía, El Pocillo, La Degollada, La Sorrueda, Las Hojas, Maspalomas, Mirador El Mulato, Mundo Aborigen, Presa de las Niñas, San Bartolomé, Santa Lucía de Tirajana: 31.III–5.IV.2019, total of 130 specimens collected from various plants including *P. dulcis*, *N. oleander*, *Phoenix canariensis*, *Yucca* sp., *Chamaecyparis* sp., *Quercus* sp., and *Cyperus* sp.

**Distribution:** Endemic Canarian species, reported to be on all islands of the archipelago [3,19].

***Tetrabrachys deserticola* (Wollaston, 1864) = *Tetrabrachys tinerfensis* (Hodgson, 1987), syn. nov.**

**Material examined:** Playa de Arinaga: 29.I.2016, 11 exx. (leg. J. Krátký); Barranco de Santa Brígida: 30.XII.2017, 1 ex. (leg. F. Pavel).

**Distribution:** Species reported to be on three islands of the Canarian archipelago, i.e., Fuerteventura, Tenerife, and Gran Canaria [3], and from Morocco [22].

**Figure 4:**
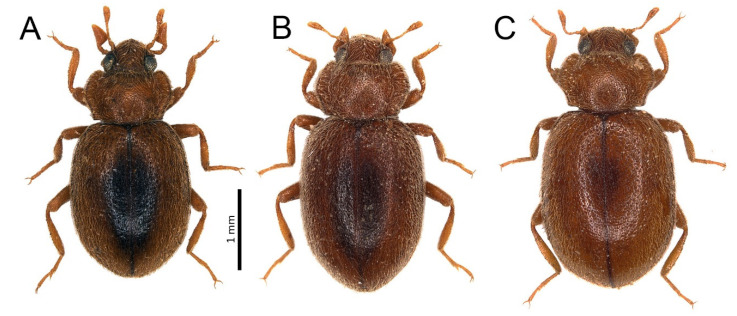
(**A–C**) Variability in the coloration of *Tetrabrachys deserticola* from Gran Canaria.

**Remarks:** According to the body coloration (especially an elliptical dark spot in the central part of elytra) and other external characters of several specimens collected on Tenerife, Hodgson [38] described a new species, *Lithophilus* (=Tetrabrachys) *tinerfensis*. All those specimens were females and, hence, male genitalia could not be examined. In the Gran Canarian material presented in this study, we found specimens of both sexes with the elytral pattern either typical of *T. tinerfensis* (Figure 4A) or *T. deserticola* (Figure 4C), or intermediate between them (Figure 4B). Male genitalia in all of these color forms did not differ from those of *T. deserticola* shown by Romanowski et al. [18]. Therefore, we propose to synonymize *Tetrabrachys tinerfensis* (Hodgson, 1987) with *Tetrabrachys deserticola* (Wollaston, 1864).

**Coccinellini Latreille, 1807**

***Adalia decempunctata* (Linnaeus, 1758)**

**Material examined:** Barranco de los Cernícalos: 28.I.2016, 1 ex. (leg. J. Krátký); Barranco Hondo: 2.IV.2019, 1 ex.; Cementerio Santa Lucía: 31.III.2019, 2 exx. from *Ficus microcarpa L.f.*; El Pocillo: 2.IV.2019, 1 ex.; Las Hojas: 1.IV.2019, 2 exx. from *Quercus* sp.; Maspalomas: 1.IV.2019, 3 exx. from *Ficus* sp., Santa Lucía de Tirajana: 3 exx. from *F.* *microcarpa.*

***Distribution:** Western Palaearctic* species [22] that probably relatively recently arrived in the Canary Islands. It was revealed for the first time by Eizaguirre [17] in the materials from the National Museum of Natural Sciences in Madrid collected on Tenerife, during the first half of 20th century. Later, it was recorded on Gran Canaria [39] and Fuerteventura [18]. Probably it was also recorded on La Palma and misidentified as *Brumus quatuorpustulatus* (sic!) (Linnaeus, 1758) by Hristova Gueorguieva [40]. This misidentified record is also quoted by Cocuzza et al. [41].

**Figure 5:**
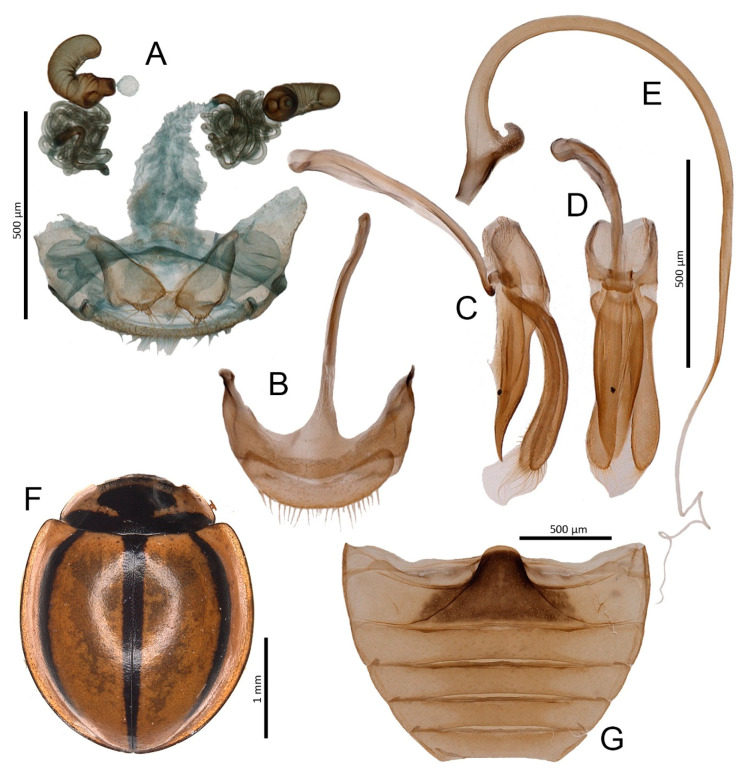
*Cheilomenes propinqua*. (**A**) Female genitalia; (**B**) Male genital segment; (**C**) Tegmen, lateral; (**D**) Tegmen, inner; (**E**) Penis lateral; (**F**) Habitus; (**G**) Abdomen, male.

***Cheilomenes propinqua* (Mulsant, 1850)** (Figure 5A–G) 

**Material examined:** Las Palmas: 4.IV.2019, 12 adults and 3 larvae collected from *Ficus* sp. and *Hibiscus* sp.; Maspalomas: 1.IV.2019, 12 adults and 4 larvae collected from *Ficus* sp. and *Hibiscus* sp.; 5.IV.2019, 27 adults and 2 larvae collected from *Phoenix canariensis*, *N. oleander*, *Ficus* sp. and *Hibiscus* sp; Vecindario: 6.IV.2019, 5 adults collected from *Bougainvillea* sp. and *Hibiscus* sp.

**Distribution:** Distributed in the Middle East, North Africa, and the Afrotropical region [22]. In May 1959, eight individuals of this species that arrived with stormy winds from Africa were collected in Santa Cruz de Tenerife [17]. The records presented here indicate that *C. propinqua* has established in the Canary Islands. New to Gran Canaria.

***Coccinella miranda* Wollaston, 1864**

**Material examined:** Mogán: 18.II.2015, 1 ex. (leg. J. Krátký); Cruz de Tejeda: 16.XII.2015, 2 exx.; Barranco de los Cernícalos: 25.I.2016, 1 ex. (leg. J. Krátký); Artenara: 29.I.2016, 1 ex. (leg. J. Krátký); Cruz de Timagada: 1.II.2016, 1 ex. (leg. J. Krátký); Santa Lucía de Tirajana: 2.II.2016, 1 ex. (leg. J. Krátký); Barranco Hondo, Barrio Coruña, Cruz de Timagada, El Gallego, El Pocillo, Juncalillo, La Degollada, La Solana, Las Hojas, Mirador de Tunte, Presa de las Niñas, Tejeda: 31.III–5.IV.2019, total of 74 specimens (65 adults, 9 larvae) collected mostly from *Pinus canariensis, P. lentiscus,* and herbaceous plants.

**Distribution:** Endemic Canarian species reported to be on all islands of the archipelago except Lanzarote [3,20].

***Coccinella septempunctata algerica* Kovář, 1977**

**Material examined:** Maspalomas: 15–16.I.2001, 2 exx. (leg. F. Pavel); Cruz Grande: 17.I.2001, 1 ex. (leg. F. Pavel); Tejeda: 18.I.2001, 1 ex. (leg. F. Pavel); Ayagaures: 17.XII.2015, 2 exx.; Cruz de Timagada: 1.II.2016, 1 ex. (leg. J. Krátký); Arucas, Ayacata, Barranco Hondo, El Gallego, El Pocillo, Firgas, Juncalillo, Las Hojas, Monte Pavón, Vega de San Mateo: 31.III–5.IV.2019, total of 59 specimens (54 adults, 2 larvae, 3 pupae) collected from herbaceous plants, *Pinus canariensis*, *Pinus nigra* J.F. Arnold, *P. lentiscus*, *Rubus* sp., and *Hedera* sp.

**Distribution:** This subspecies occurs mainly in North African countries. Reported to be on all the Canary Islands [3].

**Remarks:** Kovář [22] considers this taxon as a separate species, *C. algerica*, but according to Romanowski et al. [18] it should be treated as a subspecies of *C. septempunctata*.

***Hippodamia variegata* (Goeze, 1777)**

**Material examined:** Artenara: 29.I.2016, 1 ex. (leg. J. Krátký); Cruz de Timagada: 1.II.2016, 1 ex. (leg. J. Krátký); Barranco Hondo, El Gallego, La Degollada, Las Palmas, Maspalomas, Mirador El Mulato, Presa de las Niñas, San Bartolomé, Tejeda, Teror, Vecindario: 31.III–6.IV.2019, total of 104 specimens (83 adults, 21 larvae) collected from *N. oleander*, *Ficus* sp., *Bougainvillea* sp., *Hibiscus* sp. and herbaceous plants.

**Distribution:** Widely distributed in the Palaearctic region. It has also spread in Africa, India, North and South America, and Australia [22,42,43,44]. Nowadays *H. variegata* is common on all islands of the Canary archipelago [3], but its arrival there was probably relatively recent. Wollaston [9,10] did not record this species on any of the islands, and Uyttenboogaart was the first who found it on Gran Canaria [11], and then also on Tenerife [12].

***Myrrha octodecimguttata* (Linnaeus, 1758)**

**Material examined:** Barranco Hondo: 2.IV.2019, 3 exx. from *Pinus* sp.

**Distribution:** Palaearctic species [22], recorded in the Canaries (La Gomera), for the first time, in 1994 [16]. Recently also found on El Hierro [20]. New to Gran Canaria.

***Oenopia doublieri* (Mulsant, 1846)**

**Material examined:** Pozo Izquierdo: 5.IV.2019, 4 exx. from *Tamarix* sp.

**Distribution:** Known to be from the Mediterranean region, both in Europe and North Africa, and on the eastern islands of the Canarian archipelago (Lanzarote, Fuerteventura, Gran Canaria, and Tenerife) [3,19,22]. Recently reported to be on the Azores [45].

***Olla v-nigrum* (Mulsant, 1866)**

**Material examined:** Santa Lucía de Tirajana: 31.III.2019, 1 ex. from *O. europaea*; Vecindario: 6.IV.2019, 9 exx. Collected from *Bougainvillea* sp. and *Hibiscus* sp.

**Distribution:** Native range of this species includes North, Central and South America, and the Caribbean [46]. It has been introduced in some islands on the Pacific (Hawaii, Guam, New Caledonia, Japan), and Indian Ocean (Reunion) [47]. To our knowledge, the first records of this species in the Canary Islands are from 2014. Hristova Gueorguieva [40] in her MS thesis reported it from Tenerife and La Palma as *Harmonia axyridis* (Pallas, 1773), but the included photographs leave no doubt that it was actually *O. v-nigrum*. Them, the same erroneous data were quoted by Cocuzza et al. [41]. In 2017, *O. v-nigrum* was again recorded on Tenerife [5] and, in 2018, on Lanzarote [19]. Not previously reported to be on Gran Canaria.

**Noviini Mulsant, 1846**

***Novius cardinalis* (Mulsant, 1850)**

**Material examined:** Guía: 19.II.2015, 1 ex. (leg. J. Krátký); Los Tiles de Moya: 20.II. 2015, 1 ex. (leg. J. Krátký), 15.XII.2015, 9 exx.; Barranco de Santa Brígida: 21.II.2015, 1 ex. (leg. J. Krátký); Acusa Verde: 23.II.2015, 1 ex. (leg. J. Krátký); Cruz de Tejeda: 26.I.2016, 2 exx. (leg. J. Krátký); Valsendero: 27.I.2016, 1 ex. (leg. J. Krátký); Maspalomas: 21.VIII.2017, 1 ex. (leg. M. Piotrowska); Arucas, Cementerio Santa Lucía, El Pocillo, Las Hojas, Maspalomas, Pozo Izquierdo, San Felipe, Vecindario: 31.III–6.IV.2019, total of 29 specimens (26 adults, 3 larvae) collected from *Bougainvillea* sp., *Hibiscus* sp., *Ficus* sp., *N. oleander*, *Tamarix* sp., *Phoenix* sp., and *Juniperus* sp.

**Distribution:** Native to Australia but introduced in many regions throughout the world [22,48]. Present on all Canary Islands [3,18,19].

**Remarks:** This important biocontrol agent has for a long time been placed in the genus Rodolia Mulsant, 1850 and under this name it was also mentioned by Kovář [22]. However, Pang et al. [49] argue that Rodolia should be treated as a synonym of Novius Mulsant, 1846 and we follow their argumentation.

**Figure 6:**
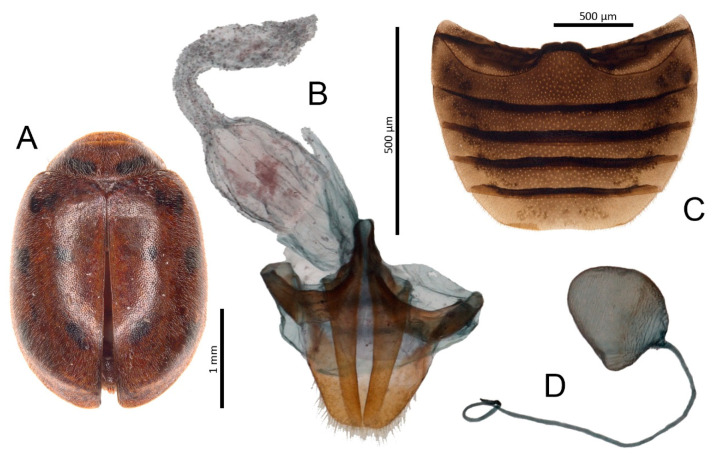
*Novius conicollis*. (**A**) Habitus; (**B**) Female genitalia; (**C**) Abdomen, female; (**D**) Spermatheca.

***Novius conicollis*Korschefsky, 1935** (Figure 6A–D) 

**Material examined:** Cruz de San Antonio: 1.IV.2019, 6 exx. (larvae) from *Pinus canariensis*; Presa de las Niñas: 31.III.2019, 4 ♀♀ from *Pinus canariensis*.

**Distribution:** Endemic Canarian species, so far reported to be on La Palma and Tenerife [3]. New to Gran Canaria.

**Remarks:** We examined only female specimens, however, the differences in the shape of coxites between *N. conicollis* and *N. canariensis* Korschefsky are distinct. In *N. conicollis*, coxites are more sclerotized with sides almost parallel. Very often the tip of ovipositor protrudes from the abdomen and can imitate the tip of the penis. In *N. canariensis,* coxites are sub-triangular and less sclerotized.

**Sticholotidini Pope, 1962**

***Pharoscymnus decemplagiatus* (Wollaston, 1857)**

**Material examined:** La Aldea de San Nicolás: 30.I.2016, 1 ex. (leg. J. Krátký); Ingenio, Barranco de Guayadeque: 2.II.2016, 1 ex. (leg. J. Krátký); Arucas, Cabo Verde, Cruz de San Antonio, Maspalomas, Pozo Izquierdo, Presa de las Niñas, Teror, Vecindario, Vega de San Mateo: 31.III–6.IV.2019, total of 42 specimens collected from *Ficus* sp, *Hibiscus* sp, *Pinus canariensis, Phoenix canariensis*, *Bougainvillea* sp., *N. oleander*, *Tamarix* sp., and unidentified Cupressaceae.

**Distribution:** Macaronesian species, reported to be on Madeira [50] and all islands of the Canary archipelago [3,18,19].

**Remarks:** Previous reports of *P. decemplagiatus* from Gran Canaria [12,51,52] concern a color form described by Uyttenboogaart [12] as *P. decemplagiatus* ssp. grancanariensis. This form has been treated by many authors, for example, [3,16,17,52] as a separate species (*P. grancanariensis*). However, Kovář [22] considers *P. grancanariensis* a synonym of *P. decemplagiatus*. In the material collected in this study, we found a continuous variability between typically colored *P. decemplagiatus* and the form ”grancanariensis”. The genital organs within this series did not differ.

### 3.2. Geographical and Historical Analysis of the Fauna of Coccinellidae in Gran Canaria

So far, 42 species of Coccinellidae have been reported to be on Gran Canaria (Table 2). Thus, Gran Canaria has currently one more reported species than Tenerife [3,5,17], which, until this study, had the richest recognized fauna of Coccinellidae on the archipelago. According to current knowledge on the general distribution of ladybirds recorded on Gran Canaria, one species (*Parexochomus bellus*) is considered to be endemic to this island, and nine other species have been considered to be endemic to the Canary archipelago. Of the seven species classified as subendemics, two (*Parexochomus quadriplagiatus* (Wollaston, 1864) and *Tetrabrachys deserticola*) have been reported to be from the Canary Islands and northwestern Africa and the remaining five from the Canary Islands and some other islands of Macaronesia. Thus, the total number of endemic and subendemic species recorded on Gran Canaria is 17, which accounts for 40% of all ladybird species known from the island.

Among the species having wider geographical ranges, only four (10%) were already known in the Canary Islands in the 19th century. The large fraction of the Gran Canarian ladybirds is composed of newcomers and presumed newcomers (21 species, 50%). Certainly, those ladybirds were colonizing the Canary Islands at various times. *Hippodamia variegata*, for example, was already detected on Gran Canaria in the 1920s [11]. Currently, it is a species well established and common throughout the archipelago [17,18,52], as well as in this study. In contrast, some other species (*Olla v-nigrum*, *Chilocorus bipustulatus, Nephus bisignatus, N. ulbrichi*) were first recorded in the Canaries just in the last years [5,19], as well as in this study.

The immigration of species to the Canary Islands is probably more intense than that to many other oceanic archipelagos, due to the proximity of the former to the mainland. Northwestern Africa is certainly a very important source of species colonizing this archipelago [79]. On the one hand, the majority of species classified in this paper as presumed newcomers most likely arrived in the Canaries from that area., and their arrival could have happened in a natural way, for example, with the help of northeastern trade winds or Saharan sandstorms. On the other hand, ladybirds mentioned in Table 2 as alien species could not appear in the Canary Islands without human mediation. All of them have come from the remote continents and have been introduced in many parts of the world as biocontrol agents. 

The detection on Gran Canaria of at least one more alien ladybird species is quite possible in the near future. *Pharoscymnus flexibilis* (Mulsant, 1853), a species of Asiatic origin, has recently been recorded on the two easternmost islands of the Canary archipelago, Fuerteventura [18] and Lanzarote [19]; on Fuerteventura, it was already widespread and common [18]. Assuming a high dispersal potential of *P. flexibilis*, its spread to further Canary Islands, first to the nearest Gran Canaria, can be expected.

Another candidate for an appearance on Gran Canaria is the well-known invasive ladybird, *Harmonia axyridis*. So far, however, there is no evidence of the establishment of this species within the Canary archipelago, and, since 2003, only single individuals have been recorded on Tenerife [17,80,81].

Unique biotas of oceanic islands, often showing high levels of endemism as compared with those in mainland regions are also highly susceptible to biotic perturbations, such as invasions by nonnative species [82,83]. The appearance of newcomers on the Canary Islands seems to be a frequent phenomenon, more frequent than on archipelagos more distant from continents and with less intense tourism. A good illustration of this is the ladybird fauna on Gran Canaria and other islands of the archipelago, which is regularly disturbed by immigrants of various origins.

## Figures and Tables

**Table 1:** Collecting sites of ladybird beetles on Gran Canaria.

Location	Coordinates
Acusa Verde	27°59′45″ N 15°41′37″ W
Agaete	28°04′01″ N 15°43′21″ W
Artenara	27°48′23″ N 15°34′58″ W
Arucas	28°07′11″ N 15°31′33″ W
Ayacata	27°55′59″ N 15°38′50″ W
Ayagaures	27°50′59″ N 15°36′32″ W
Barranco de los Cernícalos	27°58′46″ N 15°28′26″ W
Barranco de Santa Brígida	28°03′12″ N 15°28′47″ W
Barranco Hondo	28°02′36″ N 15°39′08″ W
Barrio Coruña	28°02′03″ N 15°39′54″ W
Cabo Verde	28°08′06″ N 15°35′16″ W
Cementerio Santa Lucía	27°53′52″ N 15°32′11″ W
Cruz de San Antonio	27°55′08″ N 15°41′15″ W
Cruz de Tejeda	28°00′22″ N 15°36′00″ W
Cruz de Timagada	27°57′33″ N 15°37′37″ W
Cruz Grande	27°55′47″ N 15°35′53″ W
Cueva Grande	27°59′58″ N 15°34′04″ W
Degollada de la Yegua	27°49′09″ N 15°34′44″ W
El Gallego	28°01′53″ N 15°32′46″ W
El Pocillo	28°02′43″ N 15°39′27″ W
El Rincón Barranco de la Coruña	27°58′32″ N 15°32′15″ W
Firgas	28°06′15″ N 15°33′42″ W
Fontanales	28°03′27″ N 15°36′37″ W
Guía	28°08′23″ N 15°37′59″ W
Ingenio, Barranco de Guayadeque	27°55′34″ N 15°27′45″ W
Juncalillo	28°02′18″ N 15°38′43″ W
La Aldea de San Nicolás	27°59′12″ N 15°43′17″ W
La Degollada	28°00′56″ N 15°37′55″ W
La Herradura	27°59′44″ N 15°25′37″ W
Lanzarote	28°02′11″ N 15°35′04″ W
La Solana	27°59′09″ N 15°37′41″ W
La Sorrueda	27°53′18″ N 15°32′06″ W
Las Hojas	28°02′24″ N 15°40′28″ W
Las Palmas	28°07′44″ N 15°26′03″ W
Los Tiles de Moya	28°05′34″ N 15°35′41″ W
Maspalomas	27°45′02″ N 15°35′55″ W
Mirador de Tunte	27°55′04″ N 15°34′19″ W
Mirador El Mulato	27°54′27″ N 15°41′43″ W
Mogán	27°54′45″ N 15°42′08″ W
Monte Pavón	28°03′36″ N 15°37′44″ W
Mundo Aborigen	27°48′46″ N 15°34′51″ W
Pie de la Cuesta	27°54′06″ N 15°42′29″ W
Playa de Arinaga	27°52′24″ N 15°23′03″ W
Pozo Izquierdo	27°49′34″ N 15°25′26″ W
Presa de las Niñas	27°55′41″ N 15°40′07″ W
Rosiana	27°55′17″ N 15°33′27″ W
San Bartolomé	27°55′29″ N 15°34′23″ W
San Felipe	28°08′42″ N 15°35′43″ W
Santa Lucía de Tirajana	27°54′40″ N 15°32′24″ W
Tejeda	27°59′42″ N 15°36′53″ W
Teror	28°06′26″ N 15°33′50″ W
Tufia	27°57’34″ N 15°23′05″ W
Valsendero	28°02′27″ N 15°34′40″ W
Vecindario	27°51′28″ N 15°25′52″ W
Vega de San Mateo	28°00′31″ N 15°31′59″ W

**Table 2:** Records of Coccinellidae on Gran Canaria and other islands of the Canary archipelago in the three distinguished periods. Question mark (?) before the reference [3] indicates that a given species is reported in the checklist by Oromí et al. [3], but we could not find the source data documenting this.

Species	19th Century		First Half of the 20th Century		After 1950	
	Gran Canaria	Other Islands	Gran Canaria	Other Islands	Gran Canaria	Other Islands
ENDEMIC AND SUBENDEMIC SPECIES						
Species endemic to Gran Canaria						
*Parexochomus bellus* Wollaston, 1864	[9,10]	-	[11]	-	[29] this study	-
Species endemic to the Canary Islands						
*Chilocorus canariensis* Crotch, 1874	[9]	[9]	-	[11,12]	this study	[20,28,53,54,55,56,57]
*Coccinella miranda* Wollaston, 1864	[9,58]	[8,9]	[11,12]	[12]	this study	[16,20,55,59,60,61,62]
*Nephus* (*Nephus*) *incisus* (Lindberg, 1950)	-	-	[14]	[14]	[15,52] this study	[16,18,19,20,27,54]
*Novius canariensis* Korschefsky, 1935	-	-	[13]	-	-	[20,63]
*Novius conicollis* Korschefsky, 1935	-	-	-	[13]	this study	[63]
*Scymnus* (*Mimopullus*) *cercyonides* Wollaston, 1864	-	[9,10]	[11,12,14,64]	[14]	this study	[15,20,27,54,56]
*Scymnus* (*Pullus*) *canariensis* Wollaston, 1864	[9,57]	[9]	[11,12,14,25,64]	[11,12,14,25]	[29,52] this study	[15,18,19,20,27,54,56,57,59,62,65,66]
*Scymnus* (*Pullus*) *medanensis* Eizaguirre, 2007	-	-	-	-	this study	[17,18,19]
*Stethorus tenerifensis* Fürsch, 1987	-	-	-	-	[15] this study	[15,18,19,20]
Subendemic species						
*Adalia testudinea* (Wollaston, 1854)	-	-	-	-	[17]	[67]
*Nephus* (*Nephus*) *flavopictus* (Wollaston, 1854)	[9]	[9,10]	[11]	[11,12,14]	[15,63] this study	[15,18,19,20,56,57,63,65,68,69]
*Nephus* (*Sidis*) *depressiusculus* (Wollaston, 1867)	-	-	-	-	[15]	[?3]
*Parexochomus quadriplagiatus* (Wollaston, 1864)	-	[9]	-	-	[51]	[16,18,19,28,62,63]
*Pharoscymnus decemplagiatus* (Wollaston, 1857)	-	[9,10]	[11,12]	[12]	[52] this study	[18,19,20,27,28,54,59,63,66]
*Stethorus wollastoni* Kapur, 1948	[9]	[9]	-	[12]	-	[27,63,66]
*Tetrabrachys deserticola* (Wollaston, 1864)	-	[9]	-	-	[52,70] this study	[18,38,57,62]
SPECIES WITH WIDER RANGES						
Old inhabitants of the Canary Islands						
*Coccinella septempunctata algerica* Kovář, 1977	[9,58]	[6,8,9,58]	[11,12]	[11,12]	[29,52,71] this study	[18,19,20,28,53,54,55,56,69,71]
*Oenopia doublieri* (Mulsant, 1846)	-	[9]	-	-	[17] this study	[18,19,28,63,72]
*Rhyzobius litura* (Fabricius, 1787)	[9]	8–10	[11]	[12]	this study	[19,20,56]
*Scymnus* (*Scymnus*) *rufipennis* Wollaston 1864	[9]	[9]	[14]	[14]	-	[16,63]
Presumed newcomers						
*Hippodamia variegata* (Goeze, 1777)	-	-	[11,12]	[12,73,74]	[29,52] this study	[16,18,19,20,28,54,63]
*Hyperaspis vinciguerrae* Capra, 1929	-	-	[11,12]	-	[17]	[18,28]
*Vibidia duodecimguttata* (Poda, 1761)	-	-	[11]	-	-	[?3]
*Adalia decempunctata* (Linnaeus, 1758)	-	-	-	[17]	[39] this study	[18]
*Cheilomenes propinqua* (Mulsant, 1850)	-	-	-	-	this study	[17]
*Chilocorus bipustulatus* (Linnaeus, 1758)	-	-	-	-	this study	-
*Myrrha octodecimguttata* (Linnaeus, 1758)	-	-	-	-	this study	[16,20]
*Nephus* (*Bipunctatus*) *bisignatus* (Boheman, 1850)	-	-	-	-	this study	-
*Nephus* (*Bipunctatus*) *conjunctus* (Wollaston, 1870)	-	-	-	-	[15]	[15]
*Nephus* (*Bipunctatus*) *nigricans* (Weise, 1879)	-	-	-	-	[17]	-
*Nephus* (*Nephus*) *ulbrichi* (Fürsch, 1977)	-	-	-	-	this study	-
*Novius cruentatus* (Mulsant, 1846)	-	-	-	-	[?3]	[28,56]
*Parexochomus nigripennis* (Erichson, 1843)	-	-	-	-	[52] this study	[18,19,20,55,56,63,75]
*Scymnus* (*Mimopullus*) *marinus* (Mulsant, 1850)	-	-	-	-	[15]	[15,56,57,62]
*Scymnus* (*Pullus*) *subvillosus durantae* Wollaston, 1854	-	-	-	-	this study	[15,18,19]
*Scymnus* (*Scymnus*) *nubilus* Mulsant, 1850	-	-	-	-	[15] this study	[15,18,19,20,56]
Alien species						
*Cryptolaemus montrouzieri* Mulsant, 1853	-	-	-	-	[72] this study	[18,19,20,40,63,76]
*Delphastus catalinae* (Horn, 1895)	-	-	-	-	this study	[18,19,27,28,77]
*Novius cardinalis* (Mulsant, 1850)	-	-	-	[12]	[52,63] this study	[18,19,20,27,61,63]
*Olla v-nigrum* (Mulsant, 1866)	-	-	-	-	this study	[5,19,40]
*Rhyzobius lophanthae* (Blaisdell, 1892)	-	-	-	-	[52,63,78] this study	[18,19,20,27,54,56,57,61,63,78]
